# Palliative Care in Parkinson's Disease: Role of Cognitive Behavior Therapy

**DOI:** 10.4103/0973-1075.53512

**Published:** 2009

**Authors:** Samput Mallick

**Affiliations:** Department of Psychiatry, R. G. Kar Medical College and Hospital, Kolkata, India

**Keywords:** Cognitive behaviour therapy, Palliative care, Parkinson's disease

## Abstract

**Background:**

Parkinson's disease (PD) is a chronic, progressive, neurodegenerative disorder that leads to the classic features of akinesia (encompassing hypokinesia and bradykinesia), tremor, rigidity and postural instability. Other non-motor complications include depression, fatigue, pain, and sleep disturbances. For the management of these complications, non-pharmacological techniques, such as Cognitive-behavioral therapy (CBT) can be used. This can focus on overt behavior and underlying cognitions and train the patient in coping strategies to obtain better symptom control.

**Objectives:**

To review studies on CBT as palliative care in PD patients.

**Materials and Methods:**

A survey was conducted for all available English-language studies by means of a MEDLINE search. Keywords in the searches included Parkinson's disease, palliative care, and cognitive behavior therapy. All articles that reported the cognitive behavior therapy and palliative care in a group of PD patients regardless of the method used by the researchers were identified and analyzed.

**Result and Conclusion:**

CBT has a strong evidence base for its use and has proven to be an effective treatment in management of people with chronic pain, fatigue syndrome, depression and sleep disturbances, with efficacy that lasts beyond the duration of treatment. Although PD patients suffer from these complications, there are only a few studies on administration of CBT on them. Considering its effectiveness, CBT can be used as an option for palliative care for PD patients, directed toward improving the patient's functional status, clinical disability and quality of life. Further studies are required in this area.

## INTRODUCTION

Parkinson's disease (PD), first described by Dr. James Parkinson in England in 1817, is a disorder of the brain that leads to shaking (tremors) and difficulty with walk, movement, and coordination. It develops in both men and women, mostly above 50 years of age, though it sometimes occurs in younger adults. However, it rarely affects children. PD occurs when, for reasons unknown, the nerve cells in the part of the brain called the substantia nigra which produce the neurotransmitter dopamine are slowly destroyed leading to loss of muscle movement function.[1]

PD usually begins with tremors as the first symptom in two-third of the PD patients; in others the first symptoms are usually problems with movement, or loss of smell. PD typically causes the following symptoms: tremors, stiffness, slowed movement, difficulty maintaining balance and posture [Table 1]. The PD patients may show some or all of the following symptoms: Abnormal gait, decreased arm swing, excessive salivation, feelings of depression or anxiety, general slowness of movement, lack of facial expression (*hypomimia*), lowered voice volume (*hypophonia*), small cramped handwriting (*micrographia*), stiffness of limbs, stooped posture, and tremor when resting.[2] Because the motor features of PD are so obvious, its many non-motor symptoms are easily overlooked. However, non-motor symptoms are common and troublesome and for some patients cause more disability than motor symptoms. Up to 60% of patients suffer from more than one non-motor symptom, which can be autonomic, psychiatric, or sensory. Cognitive and behavioural problems include sleep disorders, executive dysfunction, dementia, depression, anxiety, apathy, drug-induced psychosis, and disorders of impulse control. These features deserve special mention, as they are common and treatable and can lead to worsened quality of life, disability, and hospital placement.[3]

**Table 1:** Symptoms of Parkinson's disease

**Motor symptoms**
Primary
Bradykinesia and akinesia
Rigidity
Tremor
Gait problems and postural instability
Other motor symptoms
Dysphagia
Sialorrhea
Hypophonia (difficulty in speech, low sound)
Micrographia(small handwriting)
Hypomimia (reduced facial expression)
Motor initiation problems and freezing
Dystonia (involuntary contracture of muscles)
Motor complications
Motor fluctuations
End-of-dose wearing-off
Random fluctuations
Lack of response to individual levodopa dose
Early morning foot dystonia
Dyskinesia
Choreoathetoid
Dystonic
**Nonmotor symptoms**
Autonomic
Orthostatic hypotension
Drenching sweats
Drooling
Dysphagia
Constipation
Urinary frequency, Urgency
Sexual dysfunction
Sensory
Pain, paresthesia
Visual problems
Impaired sense of smell
Cognitive-behavioral
Depression
Apathy
Anxiety
Impulsive behavior
Psychosis
Dementia
Sleep disorders
Daytime sleepiness
Insomnia
Restless legs syndrome
Vivid dreams

**Figure 1:**
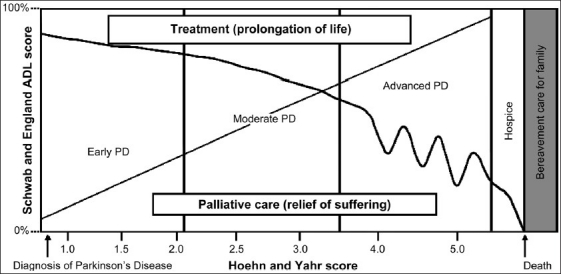
Parkinson's disease model of care (adapted from Lisette K. Bunting-Perry; Palliative Care in Parkinson's Disease: Implications for Neuroscience Nursing. J Neurosci Nurs. 2006;38(2):106-113.)

PD is a devastating disease to cause substantial morbidity and has the capacity to shorten life. Standard treatment methods prevalent today cannot cure PD, nor can alter disease progression. Hence, palliative care is needed for PD patients. Palliative care improves the quality of life of patients and their families facing the problem associated with life-threatening illness, through the prevention and relief of suffering by means of early identification and impeccable assessment and treatment of pain and other problems, physical, psychosocial and spiritual.[3] In some patients the progression of symptoms in PD may take 20 years or more, but in others, the disease progresses more quickly. There is no way to predict the disease progression. How a PD patient can perform his/her activity level can be described by Schwab and England Activities of Daily Living [Table 2]. Hoehn and Yahr scale is used to describe the stages of PD progression [Table 3]. People can live many years in the end-stage PD, it depends on many factors like their previous health status, co-existing health conditions, age, genetics, etc. Palliation for Parkinson's disease requires continued multi-disciplinary professional involvement as well as the integration and recognition of the care provided by various support groups including psychotherapists [Figure 1].

**Table 2:** Schwab and England activities of daily living

100%-Completely independent. Able to do all chores w/o slowness, difficulty, or impairment.
90%-Completely independent. Able to do all chores with some slowness, difficulty, or impairment. May take twice as long.
80%-Independent in most chores. Takes twice as long. Conscious of difficulty and slowing
70%-Not completely independent. More difficulty with chores. 3 to 4X along on chores for some. May take large part of day for chores.
60%-Some dependency. Can do most chores, but very slowly and with much effort. Errors, some impossible
50%-More dependant. Help with 1/2 of chores. Difficulty with everything
40%-Very dependant. Can assist with all chores but few alone
30%-With effort, now and then does a few chores alone of begins alone. Much help needed
20%-Nothing alone. Can do some slight help with some chores. Severe invalid
10%-Totally dependant, helpless
0%-Vegetative functions such as swallowing, bladder and bowel function are not functioning. Bedridden.

(Adapted from Gillingham FJ, Donaldson MC, eds., Third Symp. of Parkinson's Disease, Edinburgh, Scotland, EandS Livingstone, 1969, pp.152-7.)

**Table 3:** Stages of Parkinson's disease

Stage one
Signs and symptoms on one side only
Symptoms mild
Symptoms inconvenient but not disabling
Usually presents with tremor of one limb
Friends have noticed changes in posture, locomotion and facial expression
Stage two
Symptoms are bilateral
Minimal disability
Posture and gait affected
Stage three
Significant slowing of body movements
Early impairment of equilibrium on walking or standing
Generalized dysfunction that is moderately severe
Stage four
Severe symptoms
Can still walk to a limited extent
Rigidity and bradykinesia
No longer able to live alone
Tremor may be less than earlier stages
Stage five
Cachectic stage
Invalidism complete
Cannot stand or walk
Requires constant nursing care

(Hoehn and Yahr staging of parkinson's disease)

Cognitive behavioural therapy (CBT) is the term for a number of therapies which help solve problems in people's lives, such as anxiety, depression. CBT was developed from two earlier types of psychotherapy: namely Cognitive therapy, designed to change people's thoughts, beliefs, attitudes and expectations; and Behavioural therapy, designed to change how people acted. The way we think about a situation affects how we act, and in turn, our actions can affect how we think and feel. It is therefore necessary to change both the act of thinking (cognition) and behaviour at the same time.[4] This can focus on overt behaviour and underlying cognitions and train the patient in coping strategies to obtain better symptom control; hence there is a need to study on CBT as palliative care in PD patients.

## MATERIALS AND METHODS

A survey was conducted for all available English-language studies by means of a MEDLINE search. Keywords in the searches included Parkinson's disease, palliative care, and cognitive behaviour therapy. All articles that reported cognitive behaviour therapy and palliative care in a group of PD patients, regardless of the method used by the researchers, were identified and analyzed.

## RESULT

In the MEDLINE search, no articles including all three search phrases Cognitive Behaviour Therapy and Palliative care and Parkinson's disease were found. Some articles were found to use non-pharmacological therapy as Palliative care in Parkinson's disease but none of these used Cognitive Behaviour Therapy as Palliative in PD patients. The search result which included two search phrases Cognitive Behaviour Therapy and palliative care showed one article by title matching the search which was on effectiveness of brief training in Cognitive Behaviour Therapy techniques for palliative care practitioners. Search with phrases Cognitive Behaviour Therapy and Parkinson's disease shown seven articles, all dealing with depression associated with PD patients, including one study using CBT on 30 caregivers of PD patients and one comment on that study. Cole and Vaughan (2005) made the feasibility study of CBT in depression in PD patients. Dobkin and others made the clinical trial but on a small number of patients. There are, however, many studies on administration of CBT on depression of other patient group. For management of insomnia and chronic pain CBT has been found to be administered with effectiveness on other patient group.

## DISCUSSION

Non-motor symptoms occur commonly in Parkinson's disease (PD) patients across all stages of PD, and are a key determinant of quality of life.[5] These symptoms are under-reported, frequently under-recognized and under-treated. Even when identified, the common perception is many of these symptoms are untreatable. Symptoms include sleep abnormalities, fatigue, depression, mood disorders, cognitive dysfunction and pain exaggeration. Discussion of this article is limited on the use of CBT that can influence and alter some of these symptoms that such as depression, sleep disorders, and pain experience so that palliation can be done by reducing disease burden. In this article, the studies using CBT on depression of PD patients, and the studies using CBT for management of pain and sleep abnormality of other non PD patients are reviewed.

### CBT for depression in Parkinson's disease

Depression is very common in Parkinson's disease (PD), with about 40% meeting DSM-IV criteria for either major depressive disorder (20%) or dysthymia (20%). It is linked with faster progression of physical symptoms, greater cognitive decline and poorer quality of life. PD patients are not inevitably depressed, and may experience noticeable improvement in mood despite the continued presence of their medical condition. Clinicians and researchers have called for more information on how to treat depression in Parkinson's disease. CBT has been identified as a treatment of choice for a range of psychological disorders including depression with increasing application in other geriatric and medical populations. But for treatment of depression in PD (dPD), it has received little experimental attention. Cole and Vaughan (2005), in their review on the feasibility of using CBT for depression associated with Parkinson's disease, found that it as a promising option.[6]

Depressed PD patients often differ from other non- PD elderly depressed in their symptom presentation and typically have caregivers highly involved in their treatment. In order to be effective for PD patients, CBT should be tailored to their unique needs. Dobkin *et al.*, examined the effect of modified CBT to meet the needs of the depressed PD patient and incorporated a separate social support intervention for caregivers.[7] Patients received 10-14 sessions of modified individual CBT. Caregivers attended three to fourpsycho-educational sessions, occurring separately from the patient's treatment sessions. The caregivers' sessions focused on strategies for offering appropriate support, and ways to respond to the patients' negative thoughts in a targeted manner. Patients experienced a significant reduction in depressive symptoms and negative cognitions, and an increased perception of social support over the course of treatment. The Becks Depression Inventory scores suggested most of the improvement related to the cognitive dimensions of guilt, pessimism and failure. Gains were maintained at one- month follow-up.

CBT in dPD involve training in stress management, behavioural modification, sleep hygiene, relaxation techniques, and cognitive restructuring. Life stressors contributing to depressed mood need to be identified and plans to minimize stress and maximize quality of life should be formulated, both long- and short-term. Patients are helped to maintain a sense of purpose and fulfilment in their lives through meaningful activity, by adjusting their expectations of themselves. In order to maximize the amount of positive reinforcement derived from the environment; patients are encouraged to do what they found to be enjoyable. To decrease the focus on the physical condition, pleasant activities are also encouraged. Overall, behavioural strategies are adjusted to help patients maximize control in their lives. Relaxation techniques, such as diaphragmatic breathing, abbreviated progressive muscle relaxation, and guided visualization, are incorporated into the treatment. Throughout the course of treatment, patients are asked to monitor the thoughts and feelings that they had in response to stressful situations on the Automatic Thought Logs. Therapy is to be repeated in both written and oral form throughout the session.[8]

### CBT in sleep disorder in Parkinson's disease

PD patients suffer from insomnia, excessive daytime sleepiness, sleep attacks, nightmares, REM sleep behaviour disorder, periodic limb movement in sleep, restless legs syndrome and sleep apnoea syndrome. The causes of sleep disorders in PD are changes in sleep architecture, disturbances of neurotransmission, movement disturbances in sleep and medications. Natural sleep is essential for good physical and mental well-being by restoring body and mind. REM sleep helps to sustain learning, memory and mood. A sleep deprived patient is likely to develop infections, hypertension, cardiovascular disease and diabetes, depression and longer time to recover from stress.[9] CBT has emerged as an effective treatment for insomnia without evidence of adverse effects. The effects seem to last, patients sleep better than before even a year after CBT.[10]

CBT helps to change the thoughts and actions that interfere with ability to get restful sleep. The cognitive portion of CBT changes the false beliefs that affect the ability to sleep. For example, one may believe that one must get eight hours of sleep every night to function, though seven hours of sleep may be adequate. It also deals with misperceptions about the amount of time actually spent sleeping, as insomniac people often sleep more than they realize. The behavioural part of CBT helps to reprogram the sleep-wake cycle by changing specific behaviours that negatively affects sleep, such as failing to exercise or drinking beverages that contain caffeine just before bedtime.[11] CBT as an insomnia treatment usually requires four to eight 30-minute sessions with the therapist.[12] The approach works on one or more of the following levels:

- Cognitive control and psychotherapy helps to control negative thoughts and worries that disturbs sleep; and eliminates false or worrisome beliefs about sleep, e.g. the duration of sleep requirement, or idea that a single restless night will make one sick.
- Sleep restriction tries to match the time spent in bed with actual sleep requirement. Reducing the amount of time spend in bed without sleeping will actually increase desire to sleep.
- Remain passively awake by avoiding any effort to fall asleep, eliminating anxiety about falling asleep. This is called paradoxical intention.
- Stimulus control to condition a positive response to get into bed and disassociate any negative cues attached to the bedroom environment; e.g. coach the patient to use the bed only for sleep and sex.
- Sleep hygiene by developing habits that influence sleep, like, regular exercise, avoiding coffee or alcohol late in the evening, or to have a warm bath one or two hour before going to bed.
- Relaxation training such as meditation, hypnosis and muscle relaxation to reduce or eliminate the arousal that disturbs sleep.
- Bio-feedback by measuring physiological signs, like muscle tension and brain wave frequency and to control them.

### CBT in management of pain in Parkinson's disease

The prevalence of pain is 40 to 75% in patients with Parkinson disease (PD).[13] In the early stages, back and neck pain may result from stiff shoulders or neck rigidity, and leg pain may result from restless leg syndrome or dystonia. In advanced stages, pain may be caused by dyskinesia, akathisia, off period dystonia, or radicular and musculoskeletal problems. 10 to 30% patients suffer multiple sensory complaints of painful burning, stabbing, aching, itching, or tingling sensations in undefined and peculiar body regions or a vague overall sensation of tension and discomfort.[14] Pain is not only a biological response to unpleasant stimuli; but there are social and psychological factors also. The factors such as previous pain experiences, pain beliefs and fears, pain threshold, pain tolerance level and coping methods, depression and emotions (e.g. anger, sadness and anxiety) influence the experience of pain. Cognitive behavioural therapy can be beneficial in improving these factors.

A medical history should be taken and a physical examination should be done before CBT is recommended.[15] The patient is asked to describe detail pain symptoms to determine the psychosocial factors to the contribution of pain along with physical factors. The patient should also be asked to describe other symptoms being experienced in addition to pain. CBT may be conducted either in one-on-one session between a patient and a psychiatrist/psychotherapist, or in group sessions, to include more patients.[16] Typically the sessions last between one to one-and-a-half hours and are held over eight to ten weeks. They include education, skills acquisition and maintenance training to help patients to retain the information and skills learned.[17] In CBT sessions following skills may be taught to the PD patients:

- Keeping a diary to record pain sensations three times a day, and to rate the pain on a scale of 0 to 10, (0 meaning no pain and 10 meaning the worst pain possible). Physical pain and emotional pain (such as anxiety and anger) should also be recorded. It helps to identify negative patterns to be reversed by CBT.
- Pacing activities: Daily activities that cause pain (e.g., sitting at a computer) are identified. How long the activity is performed before starting of pain and when the pain disappears is recorded. Pacing activities help patients to relieve pain by reducing muscle fatigue, irritation and frustration.
- Relaxation therapy: Breathing and relaxation techniques are taught to balance the physical and emotional effects of stress and to alleviate pain.
- Cognitive therapy: Patients identify negative emotional reactions to events and learn to alter them (e.g. common emotional reactions in traffic jam may be anger and frustration, which lead to increased stress, heart rate and pain).
- Internal thoughts: Patients are taught to respond, and not to react, to stressful situations (e.g. ignoring pain and acceptance of pain).

## CONCLUSION

Cognitive behavioural therapy can benefit patients suffering from PD by decreasing psychological distress, improving pain management, increasing self-efficacy, and improving quality of life. When applied appropriately, CBT is a feasible and effective option as palliative care for Parkinson's disease patients in long term. A course of CBT is relatively short, and due to its highly structured nature, it can be provided in various formats such as software, self-help books, training material and through telephone. CBT may not be suitable for patients with complex psychiatric needs, or with learning difficulties. It addresses current problems, and focuses on specific issues, so the possible underlying causes of mental health conditions may remain unaddressed. Some patients treated with CBT may experience relapse by forgetting information or discontinuing skills learned over time, which can be reversed by follow-up therapy sessions. Larger, randomized, controlled trials are needed to further evaluate the efficacy of this kind of intervention and to identify specific mechanisms of change.[7]
